# Motifs, themes and thematic maps of an integrated *Saccharomyces cerevisiae *interaction network

**DOI:** 10.1186/jbiol23

**Published:** 2005-06-01

**Authors:** Lan V Zhang, Oliver D King, Sharyl L Wong, Debra S Goldberg, Amy HY Tong, Guillaume Lesage, Brenda Andrews, Howard Bussey, Charles Boone, Frederick P Roth

**Affiliations:** 1 Department of Biological Chemistry and Molecular Pharmacology, Harvard Medical School, Boston, MA 02115 USA; 2 Banting and Best Department of Medical Research and Department of Medical Genetics and Microbiology, University of Toronto, Toronto ON M5G 1L6, Canada; 3 Department of Biology, McGill University, Montreal PQ H3A 1B1, Canada

## Abstract

**Background:**

Large-scale studies have revealed networks of various biological interaction types, such as protein-protein interaction, genetic interaction, transcriptional regulation, sequence homology, and expression correlation. Recurring patterns of interconnection, or 'network motifs', have revealed biological insights for networks containing either one or two types of interaction.

**Results:**

To study more complex relationships involving multiple biological interaction types, we assembled an integrated *Saccharomyces cerevisiae *network in which nodes represent genes (or their protein products) and differently colored links represent the aforementioned five biological interaction types. We examined three- and four-node interconnection patterns containing multiple interaction types and found many enriched multi-color network motifs. Furthermore, we showed that most of the motifs form 'network themes' – classes of higher-order recurring interconnection patterns that encompass multiple occurrences of network motifs. Network themes can be tied to specific biological phenomena and may represent more fundamental network design principles. Examples of network themes include a pair of protein complexes with many inter-complex genetic interactions – the 'compensatory complexes' theme. Thematic maps – networks rendered in terms of such themes – can simplify an otherwise confusing tangle of biological relationships. We show this by mapping the *S. cerevisiae *network in terms of two specific network themes.

**Conclusion:**

Significantly enriched motifs in an integrated *S. cerevisiae *interaction network are often signatures of network themes, higher-order network structures that correspond to biological phenomena. Representing networks in terms of network themes provides a useful simplification of complex biological relationships.

## Background

A cellular system can be described as a web of relationships amongst genes, proteins, and other macromolecules. Proteins can interact via direct or indirect physical contact (referred to as protein-protein interactions). They can also interact genetically; for example, if a combination of mutations in two genes causes a more severe fitness defect (or death) than either mutation alone, the two genes have a synthetic sick or lethal (SSL) genetic interaction. In addition, two genes can relate to each other by transcriptional regulation, sequence homology, or expression correlation. Overlaps between different types of biological interaction have been noted previously. For example, interacting proteins are more likely to have similar expression patterns [1,2]; genes with correlated expression are more likely to be controlled by a common transcription factor [3]; and synthetic genetic interactions are more likely to occur between homologous genes [4]. These represent pairwise relationships between various types of biological interaction, however, understanding how they are organized in an integrated network remains a challenging task.

The concept of network motifs (referred to simply as 'motifs' hereafter) has been developed to describe simple patterns of interconnection in networks that occur more frequently than expected in randomized networks [5,6]. It has been proposed that network motifs represent the basic building blocks of complex networks [5-7]. Different types of network exhibit different motif profiles, providing a means for network classification [8]. The network motif concept is extensible to an integrated network of many interaction types (that is, a 'multi-color network', with interactions of each type represented by a different color). Multi-color network motifs characterize relationships between different biological interaction types within local network neighborhoods. A recent study examined network motifs in integrated cellular networks of two interaction types – transcriptional regulation and protein-protein interaction [9]. Other gene-pair relationships are also important. Correlated expression profiles may reflect common regulation or a cellular requirement for contemporaneous action. Sequence homology suggests descent from a common ancestor and therefore an increased likelihood of performing a related function. Genetic interactions describe synergistic or antagonistic consequences of mutations in two or more genes. For example, a recent systematic study [4] identified a large number of SSL interactions, revealing gene pairs in which one gene compensates for loss of the other, suggesting a functional relationship between the two gene products. Here, we describe network motifs discovered from a *Saccharomyces cerevisiae *network that integrates five types of biological interactions or relationships: protein-protein interactions, genetic interactions, transcriptional regulation, sequence homology, and expression correlation.

It has been shown for the *Escherichia coli *and *Caenorhabditis elegans *transcriptional network that subgraphs matching two types of transcriptional regulatory circuit motif – feed-forward and bi-fan – overlap with one another and form large clusters [6,10,11]. This suggests that instead of representing network "building blocks", motifs should in some cases be viewed as signatures of more fundamental higher-order structures. Here, we describe 'network themes' - recurring higher-order interconnection patterns that encompass multiple occurrences of network motifs and reflect a common organizational principle. We show that most network motifs found in the integrated *S. cerevisiae *network can be understood in terms of only a few network themes. Network themes can be tied to specific biological phenomena and may represent more fundamental network design principles. They also suggest a natural simplification of the otherwise complex set of relationships in an integrated network. We demonstrate this by providing two thematic maps of the integrated *S. cerevisiae *network.

## Results

### An integrated *S. cerevisiae *network

We constructed an integrated *S. cerevisiae *network by combining five types of biological interaction. Nodes in the network represent genes or proteins, and differently colored links represent different biological interaction types. These include: 3,060 SSL interactions derived from synthetic genetic array (SGA) analysis [4]; 40,438 protein sequence homology relationships from a genome-wide BLAST search [12]; 57,367 correlated mRNA expression relationships derived from microarray data [13]; 49,537 stable protein interactions defined by shared membership in a protein complex [14-16]; and 4,357 transcriptional regulatory interactions from a genome-wide chromatin immuno-precipitation (ChIP) study [7]. This collection of data resulted in a single integrated network involving 5,831 nodes and 154,759 links in total (for a full list see Additional data file 1).

### Three-node network motifs and corresponding themes in the integrated network

Networks of protein-protein and synthetic genetic interaction have been reported to be scale-free and 'small-world' [4,17,18]. Being a small-world network implies neighborhood clustering, where neighbors of a given node tend to interact with one another, resulting in an abundance of three-node interconnection patterns – that is, 'triangles'. In addition, relationships such as sequence homology and correlated expression are often transitive (that is, if gene A is homologous to gene B, and gene B is homologous to gene C, then gene A is often homologous to gene C). Thus, a triangle motif for each of these component subnetworks is expected. In order to find additional motifs involving multiple interaction types, we looked for frequently occurring patterns of interconnection in the integrated network, assessing their significance by comparing the observed network with appropriately randomized networks.

**Figure 1:**
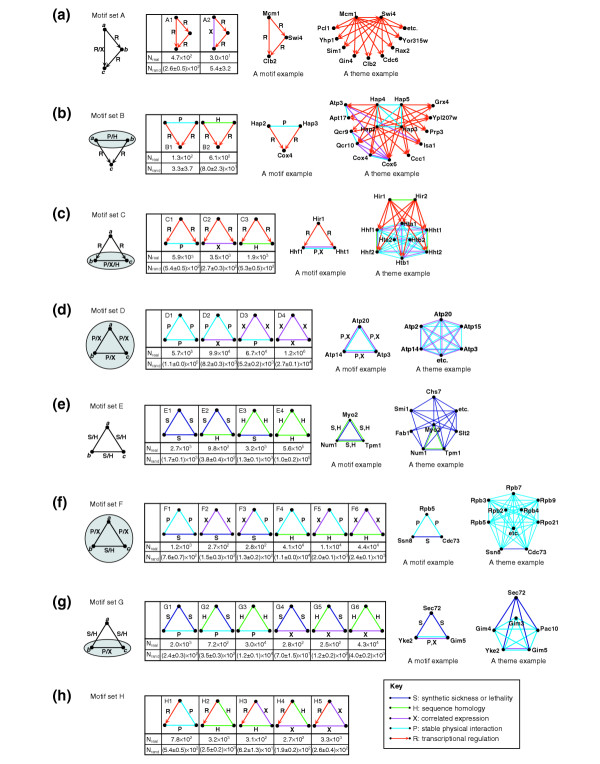
Three-node motifs and corresponding themes in the integrated *S. cerevisiae *network. **(a) **A motif corresponding to the 'feed-forward' theme; **(b) **motifs corresponding to the 'co-pointing' theme; **(c) **motifs corresponding to the 'regulonic complex' theme; **(d) **motifs corresponding to the 'protein complex' theme; **(e) **motifs corresponding to the theme of neighborhood clustering of the integrated SSL/homology network; **(f) **motifs corresponding to the 'compensatory complex members' theme; **(g) **motifs corresponding to the 'compensatory protein and complex/process' theme; **(h) **other unclassified motifs. Each of (a-g), from left to right, shows a schematic diagram unifying the collection of motifs in that set, the list of motifs with the motif statistics, a specific example of a subgraph matching one or more of these motifs, and a larger structure corresponding to the network theme. Each colored link represents one of the five interaction types according to the color scheme (bottom right). For a given motif, N_real _is the number of corresponding subgraphs in the real network, and N_rand _describes the number of corresponding subgraphs in a randomized network, represented by the average and the standard deviation. A node labeled 'etc.' signifies that the structure contains more nodes with connectivity similar to the labeled node.

We first exhaustively tested all three-node interconnection patterns defined by a single type of link between each pair of nodes (there are 50 such patterns; for a full list see Additional data file 2). Shown in Figure 1 is a list of enriched three-node network motifs, each describing a significantly (*p *< 0.001) enriched topological relationship among biological interactions of varying types in the integrated *S. cerevisiae *network. We found that most motifs can be explained in terms of higher-order structures, or network themes, which are representative of the underlying biological phenomena. We classified these motifs into seven sets (Figure 1a–g) according to the themes discussed below. There are five additional motifs that we could not classify into themes (Figure 1h). These are addressed further in the Discussion.

The first motif set contains the transcriptional feed-forward motif (Figure 1a), which has been characterized in several earlier studies of single-color networks of transcriptional regulation [5-7,11]. Because transcriptional regulation links often overlap co-expression links, we added to this set another motif composed of two genes with correlated expression that are also indirectly connected by transcriptional regulatory links through an intermediate gene. We noticed that gene triads matching the feed-forward motif in the *S. cerevisiae *network often overlap with one another to form large clusters, as in the *E. coli *and *C. elegans *transcriptional regulatory networks [6,10,11]. For example, Swi4 and its transcriptional activator Mcm1 together regulate a number of cell-cycle-related genes (Figure 1a) [19-21]. Most gene triads matching the feed-forward motif belong to such clusters, leading us to note a 'feed-forward' theme – a pair of transcription factors, one regulating the other, and both regulating a common set of target genes that are often involved in the same biological process.

The next set contains 'co-pointing' motifs, in which a target gene is regulated by two transcription factors that interact physically or share sequence homology (Figure 1b). These co-pointing motifs reflect the fact that two transcription factors regulating the same target gene are often derived from the same ancestral gene, or function as a protein complex. We found that these motifs also overlap extensively, forming a co-pointing theme, in which multiple transcription factors, connected to one another by physical interaction or sequence homology, regulate a common set of target genes. Figure 1b shows one such example, where Hap2, Hap3, Hap4 and Hap5 form the CCAAT-binding factor complex [22] which regulates common target genes, many of which are involved in carbohydrate metabolism [23].

A third set of motifs contains two targets of the same transcription factor bridged by a link of correlated expression, protein-protein interaction, or sequence homology (Figure 1c). These motifs indicate that transcriptional co-regulation is often accompanied by co-expression, membership in the same protein complex, or descent from a common ancestor [3,24], and suggest a 'regulonic complex' theme in which co-regulated proteins are often components of a complex or related by gene duplication and divergence. Illustrating this theme, six members of the histone octamer, Hhf1, Hhf2, Hht1, Hht2, Hta1 and Htb1 are all regulated by Hir1 and Hir2, histone transcriptional co-repressors that are required for periodic repression of the histone genes (Figure 1c) [25].

The fourth motif set consists of four three-node motifs each containing protein-protein interactions or correlated expression links (Figure 1d). Protein-protein interaction is known to correlate positively with co-expression [1,2], and proteins corresponding to these motifs often reside in the same complex. Thus, motifs within this set are likely to be signatures of a 'protein complex' theme. One of the many examples is the ATP synthase complex [26,27], whose members are linked extensively to one another by protein-protein interaction and correlated expression (Figure 1d).

The fifth motif set contains three-node motifs linked by SSL interaction or by sequence homology (Figure 1e). In the SSL network, neighbors of the same gene often interact with one another [4]. This translates into a triangle motif of three SSL links. Furthermore, homology relationships are often transitive (that is, if gene A is homologous to gene B, and gene B is homologous to gene C, then gene A is often homologous to gene C). These phenomena, combined with the fact that genes sharing sequence homology have an increased tendency to show SSL interaction, suggest an underlying theme of the neighborhood clustering in the integrated SSL/homology network: SSL or homology neighbors of one node tend to be linked to one another by SSL interaction or sequence homology. This theme is exemplified by Myo2 and a number of genes connected to Myo2 by SSL interaction or sequence homology (Figure 1e) [4,28,29].

The sixth motif set describes network motifs containing two nodes linked either by SSL interaction or by sequence homology, with a third node connected to each of them through protein-protein interaction or through correlated expression (Figure 1f). All three proteins (***a***, ***b ***and ***c***, as in the schematic diagram in Figure 1f) may be members of the same complex, with either ***b ***or ***c ***being sufficient to support the essential function of the complex. Proteins ***b ***and ***c ***may either reside in the complex at the same time, or be mutually exclusive (that is, competing for the same docking position in the complex). This can be generalized to a network theme of a protein complex with partially redundant or compensatory members. As one instance of this theme, both Ssn8 and Cdc73 associate with the RNA polymerase II complex [30,31], and only one of them is required for viability (Figure 1f) [4].

We found the seventh motif set particularly interesting. Motifs in this set contain two nodes linked by protein-protein interaction or correlated expression, with a third node connected to both either by SSL interaction or by sequence homology (Figure 1g). Considering previously observed correlations between protein-protein interaction and co-expression [1,2] and between SSL interaction and sequence homology [4], these motifs indicate that members of a given protein complex or biological process often have common synthetic genetic interaction partner(s) (Figure 1g). For instance, four out of the five Gim complex proteins [32] exhibit synthetic lethality with Sec72 (Figure 1g) [4]. A 'compensatory protein and complex/process' theme, in which a protein and a distinct protein complex or biological process have compensatory function, results in synthetic sickness or lethality between the protein and any member of the complex/process essential to the function of that complex/process. It is also possible for the single protein to be part of another complex/process, so that these motifs may in turn be signatures of a larger 'compensatory complexes/processes' theme, which we examine further below.

In addition to the motif sets described above, there are five motifs that we did not categorize (Figure 1h). These are especially interesting, as they may represent unknown biological phenomena (described further in the Discussion).

### Four-node network motifs corresponding to the 'compensatory complexes/processes' theme in the integrated network

**Figure 2:**
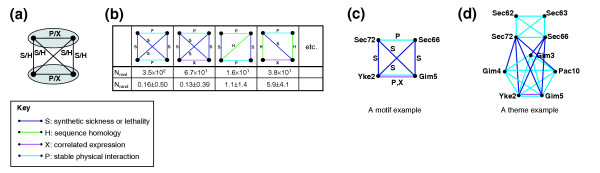
Four-node network motifs corresponding to the 'compensatory complexes/processes' theme. **(a) **A schematic diagram unifying the collection of four-node motifs corresponding to the 'compensatory complexes/processes' theme; **(b) **examples of specific four-node motifs together with the motif statistics; **(c) **a specific example of a four-node subgraph matching a few of these motifs; **(d) **the larger structure corresponding to the network theme. Each colored link represents one of the four interaction types according to the color scheme (see key). For a given motif, N_real _is the number of corresponding subgraphs in the real network, and N_rand _describes the number of corresponding subgraphs in a randomized network, represented by the average and the standard deviation.

There are over 5,000 different connected four-node interconnection patterns with each pair of nodes bridged by at most one link type. Here, we have focused on a subset of four-node patterns of particular interest. Recalling the 'compensatory protein and complex/process' theme (Figure 1g), in which a protein has compensatory function with other proteins in a complex or a process, we wondered whether there also exists a network theme corresponding to a pair of complexes/processes with compensatory function (connected to each other by many links of SSL interaction or sequence homology). We searched for all four-node interconnection patterns that would fit this 'compensatory complexes/processes' theme (there are a total of 66 such patterns – for a full list see Additional data file 3). Each pattern is composed of two pairs of nodes such that a protein-protein interaction or correlated expression link exists within each pair and SSL or sequence homology links extend between the two pairs (Figure 2). Using one thousand randomized networks to assess significance, 48 out of the 66 patterns corresponding to this theme were found to be network motifs defined by significant enrichment (*p *< 0.001) in the real network (see Figure 2 for a few examples and Additional data file 3 for a full list). This supports our hypothesis that compensatory pairs of complexes or processes are a theme in the integrated *S. cerevisiae *network. The endoplasmic reticulum (ER) protein-translocation subcomplex [33] and the Gim complex [32], connected by many SSL interactions [4], together illustrate this theme. This example also encompasses the 'compensatory protein and complex/process' theme depicted in Figure 1g, wherein multiple SSL or homology links connect Sec72 and the Gim complex.

### A thematic map of compensatory complexes

**Figure 3:**
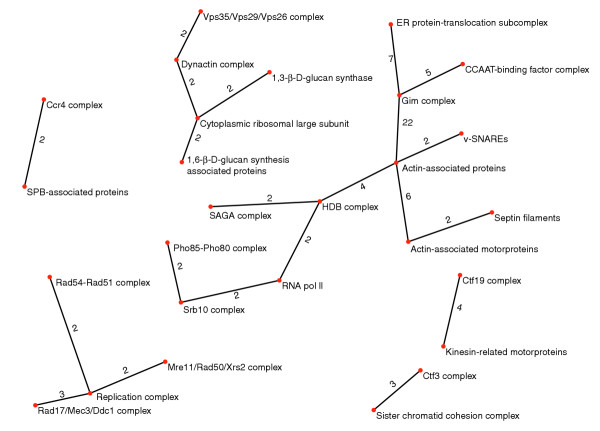
A thematic map of compensatory complexes. Here, nodes represent protein complexes, and a link is drawn between two nodes if there is a significantly large number of inter-complex SSL interactions. Links between compensatory complexes are labeled with the numbers of supporting SSL interactions.

In order to identify additional pairs of protein complexes with overlapping or compensatory function, we rendered a map of the network in terms of the 'compensatory complexes' theme. This map can also serve as a guide to 'redundant systems' within the integrated *S. cerevisiae *network, wherein two complexes provide the organism with robustness with respect to random mutation when each complex acts as a 'failsafe mechanism' for the other. To generate a thematic map of compensatory complexes, we searched for pairs of protein complexes with many inter-complex SSL interactions. For this purpose, we only considered links of protein-protein interaction and SSL interaction and reduced the original network to one in which nodes are complexes and links are SSL interactions (with multiple links allowed between a pair of 'collapsed' nodes). For each pair of protein complexes, we calculated the number of links between them and assessed the significance of enrichment (see the Materials and methods section for details). Among the 72 complexes examined (for a list of complexes see Additional data file 1), we found 21 pairs of complexes (involving 26 complexes; listed in Additional data file 4) showing significant enrichment (*p *≤ 0.05) for inter-complex SSL interactions. These compensatory complexes can be visualized as a thematic map in which each node represents a protein complex and each link bridges a pair of complexes connected by a significant number of SSL interactions (Figure 3).

### A thematic map of regulonic complexes

**Figure 4:**
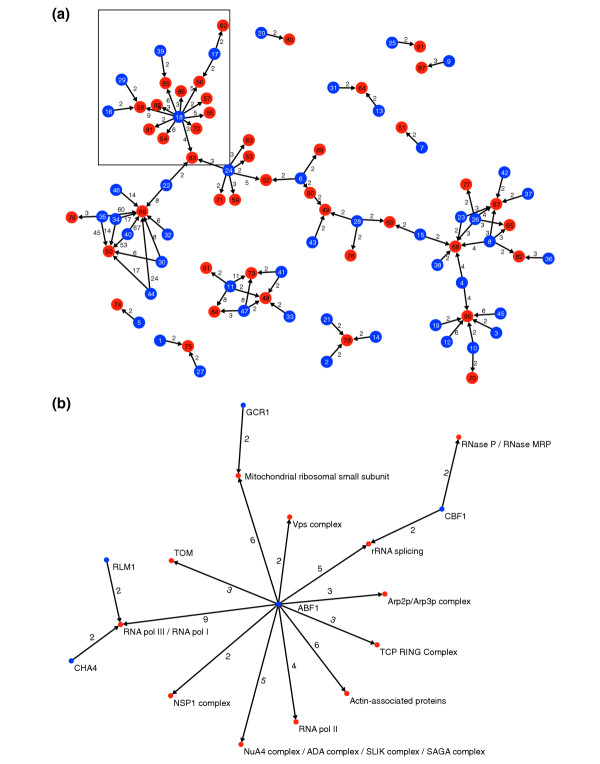
A thematic map of regulonic complexes. **(a) **Here, blue nodes represent transcription factors, red nodes represent protein complexes, and a link is drawn between a transcription factor and a protein complex if the promoters of a significantly large number of complex members are bound by the transcription factor. **(b) **An enlarged region of the regulonic complex map in (a). Links between transcription factors and the complexes they regulate are labeled with the numbers of supporting interactions in the transcription regulation network. For lists of transcription factors and complexes in the map see Additional data files 5 and 6.

Other themes depicted in Figure 1 that might be usefully exploited to generate a simplified thematic map include the 'regulonic complex' theme (Figure 1c), wherein one transcription factor (TF) regulates multiple members of a given protein complex. Such a phenomenon has been observed previously [34]. Here, we provide an automated procedure for drawing the map in terms of this network theme. To this end, we examined all possible pairings of a transcription factor with a particular protein complex (together, a 'TF-complex pair'). We reduced the integrated network of stable protein-protein interactions and transcriptional regulations to one in which nodes are either transcription factors or complexes and links indicate transcriptional regulation (with multiple links allowed between a pair of nodes). For each TF-complex pair, we calculated the number of links between them, and assessed the significance according to the probability of obtaining at least the observed number of links if each transcription factor were to choose its regulatory targets randomly. A total of 91 TF-complex pairs showed significant enrichment (*p *≤ 0.05) for transcriptional regulation links. These significant TF-complex relationships can also be viewed as a network whose nodes are transcription factors or complexes and whose links represent TF-complex pairs with significantly enriched transcriptional regulation (Figure 4a). Judging from experimental evidence, many of the links connect transcription factors and protein complexes involved in the same biological process, and complexes of related function are often connected to the same transcription factor (Figure 4b).

## Discussion

Network motifs have previously been sought in simple networks [5-7,10,11] and recently in an integrated network of transcriptional regulation and protein-protein interaction [9]. In this study, we sought network motifs in an integrated *S. cerevisiae *network with five types of biological interaction. We identified many significantly enriched motifs, which fall into several classes with distinct biological implications, revealing the interplay of different types of biological interaction in local network neighborhoods. Previously, motifs have been described as elementary building blocks of complex networks [5-7,9,11]. Here, we describe network themes – recurring higher-order interconnection patterns that encompass multiple occurrences of network motifs. We show that the abundance of most motifs in the integrated *S. cerevisiae *network can be explained in terms of a network theme.

Network themes represent a more fundamental level of abstraction that may often be preferable to network motifs for several reasons. Network motifs have been defined with artificial restrictions on the number of nodes and the specific interconnection patterns, and gene triads or tetrads corresponding to these motifs often do not exist in isolation in the network. Rather, they often overlap extensively with one another to form higher-order structures corresponding in many cases to known biological phenomena; this is supported by observations from other studies [9,10]. This phenomenon suggests that motifs are often not 'atomic' elements of the network, but are instead signatures or symptoms of more fundamental higher-order structures, or network themes. Although many motifs can be explained in terms of higher-order themes, some network motifs have an elemental function that is preserved even when that motif is embedded within a larger theme. This was demonstrated, for example, by Alon and colleagues for the coherent feed-forward loop [35].

In addition to the network themes and motifs depicted in Figure 1a–g, there are five motifs that we did not categorize (Figure 1h). Each of these motifs contains: a transcriptional regulation link, with a third node connecting to the transcription factor and its target via two stable physical interactions (motif H1); two sequence homology links (motif H2); one correlated expression link and one homology link, respectively (motif H3); one homology link and one correlated expression link, respectively (motif H4), or two correlated expression links (motif H5). Given that physical interaction links are mostly transitive, motif H1 indicates that transcription factors often co-complex with the target proteins they regulate, and suggests a mechanism of feedback regulation for transcription through protein-protein interaction. Motif H2 implies sequence homology between a transcription factor and its target, given the near transitivity of homology links. Such homology may seem unexpected but can be explained if there is frequent serial regulation of one transcription factor by another, since transcriptional factors often share homology, for example in their DNA binding domains. Motif H5 may be due simply to the overlap between transcriptional regulation links and correlated expression links, and the near transitivity of correlated expression links. The implications of motifs H3 and H4 are unclear to us; they might represent currently unknown trends in transcriptional regulatory mechanism. We hope to address some of these questions in the future by investigating the roles of genes in the subnetworks corresponding to the motifs (for example, whether the target gene in motif H2 is often a transcription factor).

Both network motifs and themes represent network characteristics that can be exploited to predict individual interactions given sometimes-uncertain experimental evidence. As has recently been shown, integration of multiple evidence types [22,36-38] can be successfully used to predict protein-protein interactions and synthetic genetic interactions, or to stratify them by confidence. In addition, the dense local neighborhood characteristic of the protein-protein interaction network can be exploited to predict protein-protein interactions [39-42]. This idea, extended to multi-color network motifs, allows us to make predictions based on topological relationships involving multiple types of links. In particular, we may predict a certain type of link between a given pair of nodes if its addition would complete a structure matching an enriched network motif. For example, two genes with a common SSL interaction partner may have increased probability of protein-protein interaction, because the addition of a protein-protein interaction link between these two genes results in a match to motif G1 (Figure 1g). Similarly, an SSL link between two genes can complete a match to motif G1 if the two genes are connected to a third gene by a protein-protein interaction link and an SSL link, respectively (Figure 1g). Such a 'two-hop physical-SSL' relationship has been recently shown to be a strong predictor of SSL interaction [38]. An interaction can also be predicted if its addition fits into a recurring network theme. For instance, there are significantly enriched SSL interactions between the ER protein-translocation subcomplex and the Gim complex (Figure 2). However, no SSL interactions have been observed between Sec62 or Sec63, two members of the ER protein-translocation subcomplex and any protein in the Gim complex because Sec62 and Sec63 were not used as queries in the SGA analysis [4]. We therefore hypothesize that Sec62 or Sec63 has SSL interactions with many members of the Gim complex.

In addition, since themes represent the network organization at the functional level, they can also be used to predict functions for genes involved in a specific theme. For example, in the feed-forward theme depicted in Figure 1a, most of the genes regulated by both Mcm1 and Swi4 are involved in control or execution of the cell cycle. We therefore hypothesize that Yor315w, a protein of unknown function, is involved in the cell cycle. More refined hypotheses can be achieved by incorporating other information such as sequence data and expression profiles. Predictions based on network themes may be robust with respect to errors in the input data, since they depend on connectivity patterns in extended network neighborhoods instead of one or very few links.

To assess whether SSL interactions involving essential genes are enriched in subgraphs matching the motifs, we counted, for each motif containing an SSL link, the fraction of subgraphs with at least one SSL interaction involving an essential gene. The results are summarized in Additional data file 2. In the SGA analysis, 11 of the 132 query genes are essential. Among the 3,060 SSL interactions, 322 of them (10.5%) involve an essential gene. Results for the network motifs are mostly consistent with this frequency of essentiality: for most motifs (E1, E2, E3, G1, G4 and G5), approximately 10% of the matching subgraphs contain SSL interactions involving an essential gene (see Additional data file 2). It is interesting, however, that subgraphs matching motifs F1 and F3 are particularly enriched with SSL interactions involving essential genes (36.4% and 24.4%, respectively). This suggests that SSL interactions within a protein complex may often involve essential genes.

Each network theme has a different biological implication, and each permits a natural simplification of the integrated network. To demonstrate this, we produced thematic maps of compensatory complexes and of regulonic complexes. The map of compensatory complexes identifies specific protein complexes with overlapping or compensatory function. Many of the links connect functionally related complexes, as supported by previous experimental evidence. For example, the replication complex, is 'genetically connected' to the Mre11/Rad50/Xrs2 complex [43], the Rad54-Rad51 complex [44], and the Rad17/Mec3/Ddc1 complex [45]. The first two function in the repair of double-strand DNA breaks [44,46] and the third is required for cell-cycle checkpoint control after DNA damage [47], both of which are associated with DNA replication. The histone deacetylase B (HDB) complex [48,49] is linked to the SAGA complex [50]; both of these affect histone acetylation and are important components of transcriptional regulation [51]. There are also some unverified but intriguing links, such as the one between the Gim complex [32] and the CCAAT-binding factor [22], which connects two seemingly unrelated complexes (Figure 3). The potential functional relationship between these complexes awaits further experimental validation.

Novel predictions for synthetic sick or lethal interactions can be made from the thematic map of compensatory complexes. Specifically, we can predict any two proteins to have an SSL interaction if they are members of two separate complexes bridged by a link in the map. There were 1,134 such protein pairs that had not been previously tested by the SGA study used to derive the compensatory complex map. We sought independent validation of these predictions among published smaller-scale studies of genetic interaction. We conservatively estimate that 10% of these pairs will have been examined for genetic interaction (note that Tong *et al*. [4], the largest systematic study to date, examined only approximately 4% of all gene pairs). Therefore, we might only hope to find approximately 113 validated pairs (10% of 1,134 predictions). Tong *et al*. [4] observed the baseline rate of SSL interaction to be 0.5%, so by chance we might expect to find fewer than one SSL interaction (0.5% of 10% of 1,134). Our literature search revealed ten gene pairs with known SSL interactions among the predictions: Arp2-Myo1 [52], Vrp1-Myo1 [53], Las17-Myo1 [54], Bem1-Myo1 [54], Rvs167-Myo1 [55], Rvs167-Myo2 [55], Smy1-Pfy1 [56], Rad50-Cdc2 [57,58], Rad54-Cdc2 [57], and Rad51-Cdc2 [58]. From this we conservatively estimate a success rate of around 9%, demonstrating the value of the thematic map in predicting new SSL interactions. Our use of the thematic map to predict genetic interactions differs from the previous prediction approach based on two-hop physical-SSL interactions [38] in that here we required a greater abundance of SSL interactions between two protein complexes than would be expected by chance, whereas previous work did not exploit the number of observed two-hop physical-SSL interactions. Furthermore, the thematic map approach has the potential to predict genetic interaction between two genes even if neither gene has any previously known SSL interactions.

In producing the thematic map of compensatory complexes, the statistical power was limited because only 4% of yeast gene pairs have been examined for synthetic genetic interactions [4]. Many compensatory complex pairs have escaped detection because too few inter-complex protein pairs have been tested for SSL to achieve statistical significance. We expect this map to grow substantially as large-scale studies of genetic interaction proceed [59]. In higher organisms for which exhaustive determination of genetic interaction is a more distant goal, we may advance our understanding more rapidly by choosing a 'scaffold' set of genes such that each known or hypothesized protein complex or pathway is represented by at least one query gene in an SSL screen.

## Materials and methods

### Constructing an integrated *S. cerevisiae *network

Synthetic genetic interactions between 132 query genes and about 5,000 array genes were obtained from a recent large-scale SGA analysis in *S. cerevisiae *[4]. Genome-wide BLAST [12] was performed using all yeast protein sequences. Pairs of proteins with *E *values of less than 10^-3 ^were considered homologous. Pearson correlation coefficients were calculated for all pairs of yeast proteins based on the Rosetta compendium microarray dataset [13]. Protein pairs with correlation coefficients larger than 0.6 were regarded as having correlated expression. Protein complexes were obtained from the MIPS [14] database and two large-scale affinity purification studies [15,16]. All pairs of proteins residing in the same complex were treated as having stable protein-protein interactions. Transcriptional regulation was inferred from the genome-wide ChIP studies of 106 yeast transcription factors [7]. If transcription factor A binds to the promoter region of gene B with a *p *value of less than 0.001, then a directed transcriptional regulatory link is assigned from A to B.

### Detecting network motifs

We enumerated all connected three-node subgraphs in the network as previously described [5]. For each interconnection pattern defined by one link between each pair of nodes, we recorded the number of subgraphs matching this pattern in the real network as well as in all randomized networks. A subgraph is considered a 'match' to the pattern if the subgraph can be transformed to the pattern by any combination of node identity permutations or link removals. The *p *value for the enrichment of an interconnection pattern is defined by the fraction of randomized networks having at least the number of matching subgraphs as the real network.

### Generating randomized networks

Different types of interactions in the integrated network were randomized independently, and then overlaid to generate a randomized multi-color network. For each interaction type, we applied a previously described method [60] to sample from an ensemble of random networks with the property that the expected degree of each node is the same as its degree in the real network. Such a method uniformly samples networks with the same degree sequence. The fugacities – parameters controlling the expected degree for each node [60] – were obtained using the multidimensional Newton-Raphson method.

Links in the network of transcription regulation are directional, originating from the transcriptional regulator and ending at the target gene. We distinguished two types of degree for each node – the in-degree (the number of links that end at the node) and the out-degree (the number of links that originate from the node). We then sampled from an ensemble of random networks [60] such that the expected in-degree and out-degree of each node in the ensemble are the same as the corresponding in-degree and out-degree, respectively, in the real network. Such a randomization procedure preserved the directionality of the transcriptional regulatory links.

Nodes in the SSL network can be divided into three mutually-exclusive categories – genes that were used as both query and array genes in the SGA analysis (denoted as 'query/array' genes), genes that were used only as query genes (denoted as 'query-only' genes), and genes that were used only as array genes (denoted as 'array-only' genes) [4]. Since an SSL link can only exist between a query gene (that is, either a 'query/array' gene or a 'query-only' gene) and an array gene (that is, either a 'query/array' gene or an 'array-only' gene) [4], we decomposed the SSL network into three sub-networks – a 'query/array↔query/array' sub-network containing only links between 'query/array' genes, a 'query-only↔query/array' sub-network containing only links between 'query-only' genes and 'query/array' genes, and a 'query↔array-only' sub-network containing only links between 'query' genes (that is, either 'query/array' or 'query-only' genes) and 'array-only' genes. When randomizing each of the three sub-networks, only links between the specified gene groups were allowed (for example, in the 'query↔array-only' sub-network, only links between 'query' genes and 'array-only' genes were allowed in the randomized network). A randomized SSL network was then generated by overlaying three such random sub-networks, one from each type. The above procedure preserved the inspection bias of the SGA method, and prohibited any link that could never be observed based on the experiment design.

### Creating the thematic map of compensatory complexes

To generate a thematic map of compensatory complexes, the integrated protein network containing SSL interaction links from the SGA analysis [4] and stable protein-protein interaction links from the MIPS complex catalog [14] was transformed to a network of protein complexes by merging multiple nodes belonging to the same protein complex into a single node. Nodes that do not belong to any known protein complex were removed, along with their associated SSL links. A few mistakes in the MIPS complex catalog were corrected, and some redundantly listed complexes were merged (for the final list of complexes see Additional data file 1). This generated a multi-graph in which multiple links are allowed between two nodes. For each pair of complexes, we recorded the number of links between them, and calculated the probability of obtaining an equal or greater number of links if each protein were to choose its SSL interaction partners randomly from all eligible proteins. Here two proteins are eligible interaction partners for each other if the pair has been tested by the SGA method [4] and both have at least one observed SSL partner in the transformed network. The nature of the SSL network, introduced from the SGA experiments, complicates the analysis, because interactions were tested only between 'query' genes and each of the 5,000 or so 'array' genes [4]. For each complex, therefore, some links originate with a query gene in the complex and end with a query gene outside the complex, some links connect a query gene within the complex and a non-query gene outside the complex, while others link a non-query gene within the complex and a query gene outside the complex. Hence, each complex has three different degree types, and the total number of links between two complexes follows a distribution corresponding to the sum of three hypergeometric distributions. The *p *values were calculated based on this composite distribution. A pair of complexes is connected in the map if the *p *value is less than 0.05 and there are two or more inter-complex SSL links.

### Creating the thematic map of regulonic complexes

The integrated protein network containing directed transcriptional regulation links from the genome-wide ChIP study (with a *p *value threshold of 0.005) [7], and stable protein-protein interaction links from the MIPS complex catalog was transformed to a network of transcription factors and protein complexes by collapsing nodes belonging to the same protein complex into a single node. Pairs of complexes that overlap by more than 50% were merged. This generates a multigraph in which multiple links are allowed between two nodes. For each TF-complex pair, we recorded the number of links between them, and calculated the probability of obtaining at least the same number of links if each node chose its interaction partners randomly. We calculated *p *values according to the cumulative hypergeometric distribution. A TF-complex pair is connected in the map if the *p *value is less than 0.05 and there are two or more regulatory links between the TF and the complex.

## Additional data files

The following supplementary tables of motifs and protein complexes are provided as Additional data files: Additional data file 1 is a zipped archive containing the five types of biological interactions in the integrated *S. cerevisiae *network as well as lists of MIPS complexes used to generated Figure 3 and Figure 4; Additional data file 2 lists all three-node interconnection patterns examined; Additional data file 3 lists all four-node interconnection patterns examined; Additional data file 4 lists all complexes in Figure 3; Additional data file 5 lists all the transcription factors in Figure 4; Additional data file 6 lists all protein complexes in Figure 4.

## Supplementary Material

Additional data file 1 A zipped archive containing the five types of biological interactions in the integrated *S. cerevisiae *network as well as lists of MIPS complexes used to generated Figure 3 and Figure 4 Click here for additional data file

Additional data file 2 All three-node interconnection patterns examined Click here for additional data file

Additional data file 3 All four-node interconnection patterns examined Click here for additional data file

Additional data file 4 All complexes in Figure 3 Click here for additional data file

Additional data file 5 All the transcription factors in Figure 4 Click here for additional data file

Additional data file 6 All protein complexes in Figure 4 Click here for additional data file
