# Supplementary material for: Motifs, themes and thematic maps of an integrated Saccharomyces cerevisiae interaction network
Source: J Biol. 2005 Jun 1;4(2):6. doi: 10.1186/jbiol23 (PMC1175995; doi:10.1186/jbiol23)
Supplement: Additional data file 2 — All three-node interconnection patterns examined [file jbiol23-s2.pdf]

Additional data file 2

A list of all three-node interconnection patterns examined

Motif ID	Interconnection pattern	*p* value	N_{real}	N_{rand}	Fraction involving essential genes (%)
A1	-R-R	<0.0002	468	$(2.6 \pm 0.5)\times 10^2$	N/A
A2	R-R-X	<0.0002	30	5.4 ± 3.2	N/A
B1	R- -P	<0.0002	131	3.3 ± 3.7	N/A
B2	R- -H	<0.0002	613	$(8.0 \pm 2.3)\times 10^1$	N/A
C1	-R-P	<0.0002	5,925	$(5.4 \pm 0.5)\times 10^2$	N/A
C2	-R-X	<0.0002	3,471	$(2.7 \pm 0.3)\times 10^2$	N/A
C3	-R-H	<0.0002	1,857	$(5.3 \pm 0.5)\times 10^2$	N/A
D1	P-P-P	<0.0002	571,461	$(1.1 \pm 0.0)\times 10^5$	N/A
D2	P-P-X	<0.0002	90,725	$(8.2 \pm 0.3)\times 10^3$	N/A
D3	P-X-X	<0.0002	67,044	$(5.2 \pm 0.2)\times 10^3$	N/A
D4	X-X-X	<0.0002	1,237,201	$(2.7 \pm 0.1)\times 10^4$	N/A
E1	S-S-S	<0.0002	2,670	$(1.7 \pm 0.1)\times 10^3$	12.5%
E2	H-S-S	<0.0002	980	$(3.8 \pm 0.4)\times 10^2$	8.1%
E3	H-H-S	<0.0002	3,160	$(1.3 \pm 0.1)\times 10^3$	10.5%
E4	H-H-H	<0.0002	563,498	$(1.0 \pm 0.2)\times 10^5$	N/A
F1	P-P-S	<0.0002	1,207	$(7.6 \pm 0.7)\times 10^2$	36.4%
F2	X-X-S	<0.0002	272	$(1.5 \pm 0.3)\times 10^2$	1.5%
F3	P-X-S	<0.0002	275	$(1.3 \pm 0.2)\times 10^2$	24.4%
F4	P-P-H	<0.0002	41,488	$(1.1 \pm 0.0)\times 10^4$	N/A
F5	P-X-H	<0.0002	10,609	$(2.0 \pm 0.1)\times 10^3$	N/A
F6	X-X-H	<0.0002	43,688	$(2.4 \pm 0.1)\times 10^3$	N/A
G1	P-S-S	<0.0002	2,046	$(2.4 \pm 0.3)\times 10^2$	8.8%
G2	P-H-S	<0.0002	720	$(3.5 \pm 0.3)\times 10^2$	24.3%
G3	P-H-H	<0.0002	29,619	$(1.2 \pm 0.1)\times 10^4$	N/A
G4	X-S-S	<0.0002	280	$(7.0 \pm 1.5)\times 10^1$	8.6%
G5	X-H-S	<0.0002	251	$(1.2 \pm 0.2)\times 10^2$	8.8%
G6	X-H-H	<0.0002	42,648	$(4.0 \pm 0.2)\times 10^3$	N/A
H1	R-P-P	<0.0002	776	$(5.4 \pm 0.5)\times 10^2$	N/A
H2	R-H-H	<0.0002	3,173	$(2.5 \pm 0.2)\times 10^3$	N/A
H3	R-H-X	<0.0002	308	$(6.2 \pm 1.3)\times 10^1$	N/A
H4	R-X-H	<0.0002	268	$(1.9 \pm 0.2)\times 10^2$	N/A
H5	R-X-X	<0.0002	3,273	$(2.6 \pm 0.4)\times 10^2$	N/A
	R-X-P	0.043	76	$(5.6 + 1.1)\times 10^1$	N/A
	R-P-X	0.432	91	$(8.9 + 1.6)\times 10^1$	N/A
	R-P-H	0.963	381	$(4.5 + 0.4)\times 10^2$	N/A
	R- -X	<0.0002	101	$(2.3 + 1.1)\times 10^1$	N/A
	R-S-X	<0.0002	58	$(1.8 + 0.6)\times 10^1$	N/A
	R-S-H	0.0046	54	$(3.1 + 0.8)\times 10^1$	N/A
	R-S-S	0.0058	49	$(2.5 + 0.8)\times 10^1$	N/A
	R-H-P	0.0166	132	$(9.3 + 1.6)\times 10^1$	N/A
	-R-S	0.0094	43	$(2.3 + 0.7)\times 10^1$	N/A
	R-R-H	0.212	41	$(3.3 + 0.9)\times 10^1$	N/A
	R-X-S	0.006	15	$4.5 + 3.1$	N/A
	R-H-S	0.0098	32	$(1.5 + 0.6)\times 10^1$	N/A
	R-R-P	0.385	8	$6.9 + 3.3$	N/A
	R-S-P	0.065	10	$5.0 + 2.7$	N/A
	R-P-S	0.199	15	$(1.1 + 0.4)\times 10^1$	N/A
	R-R-R	0.388	1	$0.5 + 0.8$	N/A
	R-R-S	0.440	1	$0.6 + 0.9$	N/A
	R- -S	1	0	$0.0 + 0.0$	N/A

For each interconnection pattern, N_{real} is the number of corresponding subgraphs in the real network, and N_{rand} describes the number of corresponding subgraphs in a randomized network, represented by the average and the standard deviation. For a significantly enriched pattern, its Motif ID corresponds to the motif identifier in Figure 1. For each motif containing an SSL link, the column ‘Fraction Involving Essential Genes (%)’ records the fraction of matching subgraphs with at least one SSL link involving an essential gene. Each three-node interconnection pattern (with nodes ***a***, ***b*** and ***c***) is described by three letters, representing the edge from node ***a*** to ***b***, the edge from node ***b*** to ***c***, and the edge from node ***c*** to ***a***, respectively. ‘S’ represents synthetic sickness or lethality; ‘H’ represents sequence homology; ‘X’ represents correlated expression; ‘P’ represents stable physical interaction; and ‘R’ represents transcriptional regulation. Because transcriptional regulation link is directed, we used ‘ ’ to represent reversed transcriptional regulation. For example, ‘ -R-X’ represents a gene triad composed of transcriptional regulation links from node ***b*** to ***a*** and from node ***b*** to ***c*** and a link of correlated expression between node ***a*** and ***c***. Note that such representation is not unique since there exist multiple ways of labeling the nodes.
