# Supplementary material for: Motifs, themes and thematic maps of an integrated Saccharomyces cerevisiae interaction network
Source: J Biol. 2005 Jun 1;4(2):6. doi: 10.1186/jbiol23 (PMC1175995; doi:10.1186/jbiol23)
Supplement: Additional data file 3 — All four-node interconnection patterns examined [file jbiol23-s3.pdf]

Additional data file 3

A list of all four-node interconnection patterns examined

Interconnection pattern	*p*-value	N_{real}	N_{rand}
X, P, H, ∅, ∅, ∅	<0.001	14,087,024	$(9.7 \pm 0.2)\times10^6$
P, P, H, ∅, ∅, ∅	<0.001	11,162,140	$(9.0 \pm 0.2)\times10^6$
X, X, H, ∅, ∅, ∅	<0.001	5,222,901	$(1.7 \pm 0.1)\times10^6$
X, P, S, ∅, ∅, ∅	<0.001	1,134,424	$(6.1 \pm 0.5)\times10^5$
P, P, S, ∅, ∅, ∅	0.016	733,571	$(6.4 \pm 0.4)\times10^5$
P, P, H, H, ∅, ∅	<0.001	478,222	$(1.3 \pm 0.1)\times10^5$
X, X, S, ∅, ∅, ∅	<0.001	341,039	$(1.0 \pm 0.2)\times10^5$
X, X, H, H, ∅, ∅	<0.001	289,017	$(2.1 \pm 0.2)\times10^4$
X, P, H, ∅, H, ∅	<0.001	253,580	$(5.6 \pm 0.4)\times10^4$
X, P, H, H, ∅, ∅	<0.001	227,144	$(7.5 \pm 0.5)\times10^4$
P, P, H, ∅, ∅, H	<0.001	29,813	$(1.2 \pm 0.1)\times10^4$
P, P, S, S, ∅, ∅	<0.001	26,940	$(3.0 \pm 0.5)\times10^3$
X, P, S, S, ∅, ∅	<0.001	23,040	$(2.3 \pm 0.7)\times10^3$
X, P, H, ∅, ∅, H	<0.001	21,445	$(1.0 \pm 0.1)\times10^4$
X, X, H, ∅, ∅, H	<0.001	11,919	$(1.4 \pm 0.2)\times10^3$
P, P, H, S, ∅, ∅	<0.001	11,805	$(6.7 \pm 1.0)\times10^3$
P, P, H, H, H, ∅	<0.001	9,832	$(1.7 \pm 0.2)\times10^3$
X, X, H, H, H, ∅	<0.001	8,941	$(2.2 \pm 0.5)\times10^2$
X, P, H, H, H, ∅	<0.001	7,562	$(1.5 \pm 0.2)\times10^3$
X, P, H, ∅, S, ∅	<0.001	6,116	$(2.6 \pm 0.6)\times10^3$
X, X, H, H, H, H	<0.001	6,063	4.2 ± 2.8
X, P, S, ∅, S, ∅	<0.001	3,669	$(9.8 \pm 3.0)\times10^2$
X, P, H, S, ∅, ∅	<0.001	3,085	$(1.3 \pm 0.3)\times10^3$
P, P, H, ∅, ∅, S	<0.001	2,836	$(1.6 \pm 0.2)\times10^3$
P, P, H, H, H, H	<0.001	2,650	$(3.1 \pm 0.9)\times10^1$
X, P, H, H, H, H	<0.001	2,146	$(3.0 \pm 0.9)\times10^1$
X, P, H, ∅, ∅, S	<0.001	2,039	$(1.3 \pm 1.7)\times10^3$
X, X, S, S, ∅, ∅	0.004	1,400	$(4.7 \pm 2.7)\times10^2$
X, X, H, S, ∅, ∅	<0.001	1,313	$(3.8 \pm 1.6)\times10^2$
P, P, S, ∅, ∅, S	<0.001	1,141	$(5.4 \pm 1.8)\times10^1$
P, P, S, S, S, ∅	<0.001	903	8.8 ± 5.4
P, P, H, H, S, ∅	<0.001	507	$(9.3 \pm 2.1)\times10^1$
X, X, H, ∅, ∅, S	<0.001	464	$(1.7 \pm 0.4)\times10^2$
X, P, S, ∅, ∅, S	<0.001	442	$(3.5 \pm 1.4)\times10^1$
P, P, S, S, S, S	<0.001	346	0.16 ± 0.50
X, P, S, S, S, ∅	<0.001	305	7.9 ± 5.3
X, P, H, H, S, ∅	<0.001	244	$(4.0 \pm 1.5)\times10^1$
P, P, H, S, ∅, S	<0.001	186	$(3.1 \pm 1.1)\times10^1$
X, P, H, ∅, S, S	<0.001	88	$(1.9 \pm 1.1)\times10^1$
X, P, S, S, S, S	<0.001	67	0.13 ± 0.39
P, P, H, S, ∅, H	<0.001	64	$(1.7 \pm 0.8)\times10^1$
X, P, H, S, H, ∅	0.001	51	$(1.6 \pm 0.7)\times10^1$
X, P, H, S, ∅, H	<0.001	38	5.9 ± 4.1
P, P, H, H, H, S	<0.001	29	1.2 ± 1.3
X, P, H, S, ∅, S	<0.001	27	5.2 ± 3.5
X, X, S, ∅, ∅, S	<0.001	25	3.6 ± 2.9
X, X, H, H, S, ∅	0.002	24	4.6 ± 3.6
X, X, H, S, ∅, S	0.001	16	2.3 ± 2.6
P, P, H, S, S, ∅	<0.001	16	1.1 ± 1.4
P, P, H, H, S, S	<0.001	10	0.42 ± 0.74
X, X, H, S, ∅, H	<0.001	8	0.28 ± 0.69
X, P, H, H, S, S	<0.001	7	0.30 ± 0.62
P, P, H, S, S, S	<0.001	3	0.09 ± 0.32
X, X, S, S, S, ∅	0.215	2	0.9 ± 1.4
X, P, H, H, H, S	0.102	2	0.51 ± 0.79
X, P, H, S, H, S	0.035	1	0.04 ± 0.20
X, X, S, S, S, S	1.000	0	0.02 ± 0.15
X, X, H, S, S, ∅	1.000	0	0.02 ± 0.15
X, X, H, S, S, S	1.000	0	0.002 ± 0.045

Additional data file 3 *(continued)*

A list of all four-node interconnection patterns examined

Interconnection pattern	*p*-value	N_{real}	N_{rand}
X, X, H, S, S, H	1.000	0	0.0 ± 0.0
X, X, H, H, S, S	1.000	0	0.02 ± 0.15
X, X, H, H, H, S	1.000	0	0.02 ± 0.15
X, P, H, S, S, Ø	1.000	0	0.36 ± 0.75
X, P, H, S, S, S	1.000	0	0.05 ± 0.22
X, P, H, S, S, H	1.000	0	0.001 ± 0.032
P, P, H, S, S, H	1.000	0	0.004 ± 0.063

For each interconnection pattern, N_{real} is the number of corresponding subgraphs in the real network, and N_{rand} describes the number of corresponding subgraphs in a randomized network, represented by the average and the standard deviation. Each four-node interconnection pattern (with nodes ***a***, ***b***, ***c*** and ***d***) is described by six letters, representing the edge between node ***a*** and ***b***, the edge between node ***c*** and ***d***, the edge between node ***a*** and ***c***, the edge between node ***a*** and ***d***, the edge between node ***b*** and ***c***, and the edge between node ***b*** and ***d***, respectively. 'S' represents synthetic sickness or lethality; 'H' represents sequence homology; 'X' represents correlated expression; and 'P' represents stable physical interaction. 'Ø' signifies that no link is present between the corresponding nodes. Note that such representation is not unique since there exist multiple ways of labeling the nodes.
