# Supplementary material for: Motifs, themes and thematic maps of an integrated Saccharomyces cerevisiae interaction network
Source: J Biol. 2005 Jun 1;4(2):6. doi: 10.1186/jbiol23 (PMC1175995; doi:10.1186/jbiol23)
Supplement: Additional data file 4 — All complexes in Figure 3 [file jbiol23-s4.pdf]

Additional data file 4

A list of all complexes in Figure 3

Complex	Members
Gim complex	Yke2, Gim3, Pac10, Gim4, Gim5
CCAAT-binding factor complex	Hap3, Hap4, Hap2, Hap5
Actin-associated proteins	Tpm1, Glk1, Bud6, Aip1, Pan1, Dld2, Crn1, Arp2, Cap2, Cap1, Sac6, Abp1, Sla1, Vrp1, Srv2, Sla2, Tpm2, Oye2, Bem1, Las17, Rvs167, Pfy1, Cof1, Twf1, Arc40
ER protein-translocation subcomplex	Sec66, Sec62, Sec72, Sec63
Ctf19 complex	Okp1, Ctf19, Mcm21
Kinesin-related motorproteins	Cik1, Cin8, Kip2, Kar3, Lin1, Kip3, Cog7, Kip1
Dynactin complex	Jnm1, Arp1, Nip100
Cytoplasmic ribosomal large subunit	Rpl19a, Rpl8b, Rpl11b, Rpp0, Rpl6b, Rpl16a, Rpl26a, Rpl7b, Rpl17b, Rpl23b, Rpl18b, Rpl19b, Rpl36a, Rpl14b, Rpl4a, Rpl1b, Rpl31a, Rpp2a, Rpl42b, Rpl22a, Rpl15a, Rpl28, Rpl41a, Rpl42a, Rpp1a, Rpl30, Rpl4b, Rpl24a, Rpl5, Rpl12a, Rpl11a, Rpl39, Rpl33b, Rpl9a, Rpl13a, Rpl34a, Rpl20a, Rpl7a, Rpl20b, Rpl35a, Rpl3, Rpl40b, Rpl21a, Rpl10, Rpl38, Rpl26b, Rpl17a, Rpp1b, Rpl37a, Rpl33a, Rpl14a, Rpl12b, Rpl43a, Rpl6a, Rpl2a, Rpp2b, Rpl13b, Rpl18a, Rpl27a, Rpl40a, Rpl31b, Rpl23a, Rpl25, Rpl9b, Rpl16b, Rpl32, Rpl41b, Rpl1a, Rpl24b, Rpl35b, Rpl37b, Rpl8a, Rpl22b, Rpl15b, Rpl29, Rpl36b, Rpl43b, Rpl34b, Rpl27b, Rpl21b, Rpl2b
Vps35/Vps29/Vps26 complex	Pep8, Vps35, Vps29
HDB complex	Sap30, Rpd3, Pho23, Sin3
SAGA complex	Spt7, Spt20, Taf6, Gcn5, Spt8, Taf5, Spt3, Ada2, Tra1, Taf12, Ngg1, Hfi1, Sgf73, Sgf29, Taf10, Taf9
RNA polII	Rpb5, Rpb2, Rpc10, Rpb3, Cdc73, Rpb11, Rpb4, Rpb8, Rpo26, Rpb7, Rpb10, Rpo21, Rpb9
Ccr4 complex	Ccr4, Cdc39, Dhh1, Dbf2, Not3, Caf16, Mob1, Not5, Caf4, Mot2, Caf17, Pop2, Cdc36
SPB-associated proteins	Stu2, Cik1, Kar3, Jnm1, Ame1, Spc19, Nuf2, Arp1, Tid3, Spc34, Dyn1, Kar5, Bik1, Stu1
Rad54-Rad51 complex	Rad54, Rad51
Replication complex	Dpb3, Orc5, Pol2, Mcm6, Cdc54, Orc1, Cdc45, Cdc46, Mcm3, Pol32, Dpb11, Cdc2, Dbf4, Cdc7, Mcm2, Cdc47, Orc3, Orc4, Orc6, Hys2, Orc2, Dpb2
Rad17/Mec3/Ddc1 complex	Rad17, Mec3, Ddc1
Sister chromatid cohesion complex	Scc2, Mcd1, Smc3, Spo69, Smc1, Irr1
Ctf3 complex	Mcm22, Mcm16, Ctf3
Mre11/Rad50/Xrs2 complex	Mre11, Rad50, Xrs2
Actin-associated motorproteins	Myo4, Myo5, Smy2, Myo1, Smy1, Myo2, Myo3
Septin filaments	Spr28, Shs1, Spr3, Cdc10, Cdc3, Cdc12, Cdc11
Pho85-Pho80 complex	Pho85, , Pho80
Srb10 complex	Ssn2, Ssn3, Srb8, Ssn8
1,3-β-D-glucan synthase	Fks1, Rho1, Gsc2
v-SNAREs	Gos1, Nyv1, Snc1, Bet1, Bos1, Sec22, Vti1, Snc2
1,6-β-D-glucan synthesis associated proteins	Skn1, Kre6
