# Supplementary material for: Motifs, themes and thematic maps of an integrated Saccharomyces cerevisiae interaction network
Source: J Biol. 2005 Jun 1;4(2):6. doi: 10.1186/jbiol23 (PMC1175995; doi:10.1186/jbiol23)
Supplement: Additional data file 5 — All the transcription factors in Figure 4 [file jbiol23-s5.pdf]

Additional data file 5

A list of all the transcription factors in Figure 4 shown as blue nodes

Index	TF
1	Ino2
2	Rtg3
3	Pho2
4	Swi4
5	Gcn4
6	Fkh1
7	Adr1
8	Swi6
9	Bas1
10	Met4
11	Hap4
12	Hir1
13	Sum1
14	Leu3
15	Ndd1
16	Cha4
17	Cbf1
18	Abf1
19	Mth1
20	Zap1
21	Arg80
22	Sfp1
23	Mcm1
24	Reb1
25	Rox1
26	Mbp1
27	Ino4
28	Fkh2
29	Rlm1
30	Rgm1
31	Uga3
32	Smp1
33	Hap5
34	Yap5
35	Rap1
36	Rfx1
37	Mot2
38	Stb1
39	Gcr1
40	Fhl1
41	Hap3
42	Sok2
43	Gat1
44	Gat3
45	Hir2
46	Pdr1
47	Hap2
