# Supplementary material for: Motifs, themes and thematic maps of an integrated Saccharomyces cerevisiae interaction network
Source: J Biol. 2005 Jun 1;4(2):6. doi: 10.1186/jbiol23 (PMC1175995; doi:10.1186/jbiol23)
Supplement: Additional data file 6 — All protein complexes in Figure 4 [file jbiol23-s6.pdf]

Additional data file 6

A list of all protein complexes in Figure 4 shown as red nodes

Index	Complex	Members
48	Cytochrome c oxidase	Cox12, Cox7, Cox4, Cox1, Cox8, Cox13, Cox2, Cox5a, Cox3, Cox9, Cox6
49	Ndc80 protein complex / Dynactin complex / SPB associated proteins	Ame1, Jnm1, Cik1, Spc19, Stu2, Spc24, Spc25, Arp1, Tid3, Kar3, Nuf2, Spc34, Kar5, Nip100, Dyn1, Bik1, Stu1
50	Cytoplasmic ribosomal small subunit	Rps28a, Rps7b, Rps14a, Rps9b, Rps23a, Rps15, Rps26a, Rps12, Rps31, Rps26b, Rps0a, Rps19b, Rps7a, Rps22b, Rps23b, Rps16a, Rps19a, Rps17a, Rps11b, Rps10b, Rps18b, Rps8a, Rps5, Rps29b, Rps6a, Rps11a, Rps3, Rps6b, Rps0b, Rps8b, Rps2, Rps25b, Rps13, Rps28b, Rps1a, Rps25a, Rps27a, Rps29a, Rps21a, Asc1, Rps1b, Rps20, Rps22a, Rps24a, Rps14b, Rps30a, Rps4a, Rps21b, Rps17b, Rps30b, Rps18a, Rps27b, Rps24b, Rps10a, Rps16b, Rps4b, Rps9a
51	Srb10p complex	Ssn2, Ssn3, Srb8, Ssn8
52	19/22S regulator	Rpt2, Rpn6, Rpt4, Rpn11, Rpn3, Rpn8, Rpt1, Rpt3, Rpn7, Nas6, Rpn9, Rpn12, Rpt5, Rpt6, Rpn10, Rpn5, Rpn1, Rpn2
53	COPII	Sec24, Erv25, Sec13, Sec16, Sec31, Emp24, Erp2, Sec23, Erv46, Erp1, Sar1
54	Actin-associated proteins	Tpm1, Aip1, Bud6, Glk1, Pan1, Dld2, Crn1, Cap1, Cap2, Arp2, Sac6, Srv2, Vrp1, Sla1, Abp1, Sla2, Las17, Bem1, Oye2, Tpm2, Rvs167, Pfy1, Cof1, Twf1, Arc40
55	NuA4 complex / ADA complex / SLIK complex / SAGA complex	Spt20, Spt7, Gcn5, Taf6, Spt8, Hfi1, Taf5, Ada2, Tra1, Spt3, Taf12, Sgf73, Sgf29, Esa1, Taf9, Taf10, Ngg1
56	rRNA splicing	Ifh1, Nop1, Utp8, Utp11, Utp4, Utp5, Pop2, Pop1, Nsr1, Npl3, Utp10, Utp7, Sbp1, Rrp3, Gar1, Drs1, Utp6, Prp12, Rnt1, Rat1, Srd1, Snm1, Utp9, Utp13
57	NSP1 complex	Nic96, Nup49, Nsp1, Nup57
58	RNA pol III / RNA pol I	Rpb5, Rpc10, Rpa43, Rpa190, Rpc25, Rpa135, Rpc19, Rpa14, Rpc40, Rpc31, Rpa49, Rpa12, Rpc34, Rpo31, Rpa34, Ret1, Rpb8, Rpo26, Rpb10, Rpc82
59	H^+-transporting ATPase, vacuolar	Vma6, Vma10, Tfp1, Vma7, Vma4, Vma2, Vph1, Vma5, Cup5, Stv1, Vma13, Ppa1, Tfp3, Vma8
60	NOT/CCR4 complex	Cdc39, Ccr4, Dhh1, Not3, Dbf2, Caf16, Mob1, Not5, Caf4, Mot2, Caf17, Pop2, Cdc36
61	Other respiration chain complexes	Sdh1, Fum1, Cit1, Yor356w, Ald4, Nde1, Ypr004c, Ygr207c, Mdh1, Ndi1, Nde2, Gut2, Cyb2, Dld1
62	RNase P / RNase MRP	Rpp1, Rpr2, Pop6, Pop3, Pop5, Pop7, Pop1, Pop4, Pop8
63	AP-1 complex	Apm1, Apl4, Aps1, Apl2
64	TFIIH / Kin28p complex / NEF3 complex	Ccl1, Rad3, Ssl1, Kin28, Tfb2, Tfb3, Ssl2, Tfb1, Tfb4, Rad2
65	Sister chromatid cohesion complex	Scc2, Smc3, Mcd1, Spo69, Irr1, Smc1
66	Cytoplasmic ribosomal large subunit	Rpl8b, Rpl19a, Rpl11b, Rpp0, Rpl6b, Rpl16a, Rpl26a, Rpl7b, Rpl17b, Rpl23b, Rpl18b, Rpl19b, Rpl36a, Rpl14b, Rpl4a, Rpl1b, Rpl31a, Rpp2a, Rpl42b, Rpl22a, Rpl15a, Rpl28, Rpl41a, Rpl42a, Rpp1a, Rpl30, Rpl4b, Rpl24a, Rpl5, Rpl12a, Rpl11a, Rpl39, Rpl33b, Rpl34a, Rpl13a, Rpl9a, Rpl20a, Rpl7a, Rpl20b, Rpl35a, Rpl3, Rpl40b, Rpl21a, Rpl10, Rpl38, Rpl26b, Rpl17a, Rpp1b, Rpl37a, Rpl33a, Rpl14a, Rpl12b, Rpl43a, Rpl6a, Rpl2a, Rpp2b, Rpl13b, Rpl18a, Rpl27a, Rpl40a, Rpl31b, Rpl23a, Rpl25, Rpl9b, Rpl16b, Rpl32, Rpl41b, Rpl1a, Rpl35b, Rpl24b, Rpl37b, Rpl8a, Rpl22b, Rpl29, Rpl15b, Rpl36b, Rpl34b, Rpl43b, Rpl27b, Rpl21b, Rpl2b
67	Replication initiation complex / ORC / DNA pol delta (III) / pre-RC / Replication complex	Sld3, Cdc7, Dpb3, Orc5, Pol2, Mcm6, Cdc45, Cdc54, Orc1, Cdc46, Mcm3, Cdc6, Dbf4, Pol32, Dpb11, Mcm2, Cdc47, Orc3, Orc6, Orc4, Hys2, Orc2, Dpb2, Cdc2
68	Cdc28p complexes	Cln1, Clb1, Clb6, Cln3, Cln2, Clb4, Clb3, Clb2, Clb5, Cdc28
69	H^+-ATPase, plasma membrane	Pmp1, Pmp2, Pma1, Pma2
70	Isocitrate dehydrogenase	Idh2, Idh1
71	v-SNAREs	Nyv1, Gos1, Bos1, Bet1, Snc1, Sec22, Snc2, Vti1
72	Arp2p/Arp3p complex	Arc15, Arp3, Arc35, Arc18, Arc19, Arp2
73	F0/F1 ATP synthase	Atp5, Atp20, Atp7, Atp4, Atp14, Atp15, Atp3, Tim11, Atp18, Atp17, Oli1, Atp19, Aap1, Atp6, Atp2, Inh1, Atp16, Atp1

Additional data file 6 *(continued)*

A list of all protein complexes in Figure 4 shown as red nodes

Index	Complex	Members
74	Arginine-specific carbamoylphosphate synthase	Cpa1, Cpa2
75	Fatty acid synthetase, cytoplasmic	Fas2, Fas1
76	Kinesin-related motorproteins	Kip3, Cik1, Cog7, Kip2, Cin8, Kip1, Kar3, Lin1
77	Replication factor A complex	Rfa1, Rfa2, Rfa3
78	Mitochondrial ribosomal large subunit	Mrpl31, Img1, Mrpl13, Mrpl27, Mrpl35, Mrpl33, Img2, Mrpl10, Mrpl7, Mrpl32, Rml2, Ypl183w-a, Mrpl3, Mrpl24, Mrpl20, Mrpl17, Mrpl39, Yml6, Mrpl36, Mrp7, Mrpl8, Ygl068w, Mrpl22, Mrpl37, Ydr115w, Mrpl25, Mrpl15, Mrpl11, Mrpl1, Mrp49, Mrpl50, Mrpl38, Mrpl4, Mrpl19, Mrpl9, Mrpl51, Mrp20, Mrpl49, Mrpl16, Mrpl40, Mrpl28, Mrpl44, Mrpl6, Mrpl23
79	eEF1	Tef4, Efb1, Cam1, Hbs1, Tef2, Tef1
80	TRAPP (Transport Protein Particle) complex	Bet3, Gsg1, Trs20, Trs33, Trs130, Kre11, Trs31, Trs120, Trs23, Bet5
81	Vps4p ATPase complex	Vps24, Vps4, Snf7
82	Ribonucleoside-diphosphate reductase	Rnr4, Rnr1, Rnr3, Rnr2
83	RNA pol II	Rpb2, Rpb5, Rpc10, Rpb3, Rpb11, Cdc73, Rpb4, Rpb8, Rpo26, Rpb7, Rpb10, Rpo21, Rpb9
84	Cytochrome bc1 complex	Qcr6, Cor1, Qcr9, Rip1, Qcr7, Cob, Qcr8, Cyt1, Qcr2, Qcr10
85	Mitochondrial ribosomal small subunit	Mrps5, Rsm25, Mrp13, Rsm19, Mrps18, Rsm22, Rsm10, Rsm18, Nam9, Rsm23, Mrp17, Mrp51, Rsm27, Mrps17, Rsm24, Mrp10, Mrps28, Mrp1, Ymr31, Mrps35, Mrp2, Ppe1, Rsm26, Mrps9, Mrp4, Var1, Rsm7, Mrps8, Mrps16, Mrp21, Pet123
86	TOM (transport across the outer membrane) complex	Tom20, Tom5, Tom70, Tom22, Tom40, Tom6, Tom7, Tom37, Tom71
87	Glycine decarboxylase	Gcv1, Lpd1, Gcv2, Gcv3
88	TCP RING Complex	Cct5, Cct7, Tcp1, Cct6, Cct4, Cct2, Cct3, Cct8
89	Sister chromatid separation complex	Esp1, Pds1, Src1
90	Nucleosomal protein complex	Hhf1, Hta2, Hta1, Hht1, Hhf2, Hht2, Htb1, Htb2
91	Ume6/Ime1 complex	Ume6, Ime1
